# Framing resilience linked to parental ethnic-racial socialization as hidden: A hidden resilience conceptual framework

**DOI:** 10.1111/spc3.12984

**Published:** 2024-07-09

**Authors:** Juan Del Toro, Donte Bernard, Richard M. Lee, Emma K. Adam

**Affiliations:** 1 University of Minnesota-Twin Cities, Minneapolis, Minnesota, USA; 2 University of Missouri-Columbia, Columbia, Missouri, USA; 3 Northwestern University, Evanston, Illinois, USA

**Keywords:** ethnic-racial discrimination, ethnic-racial socialization, resilience

## Abstract

Parental ethnic-racial socialization is a source of adolescents’ resilience against ethnic-racial discrimination. Recent meta-analyses have documented the *promotive* aspects of ethnic-racial socialization (i.e., how ethnic-racial socialization is directly related with adolescents’ adjustment regardless of their discrimination experiences). However, extant empirical studies have produced conflicting results about the *protection* or moderating role of ethnic-racial socialization, with studies suggesting that ethnic-racial socialization buffers, exacerbates, or does not moderate the impacts of ethnic-racial discrimination. We offer a reconceptualization of existing studies’ findings and draw from existing theories to propose *Hidden Resilience* as a new conceptual framework that highlights how resilience and the positive benefits linked to ethnic-racial socialization may not be noticeable when studies use psychosocial measures but is rather hidden “underneath the skin.” Conversations about racism may momentarily feel uncomfortable, upsetting, or stressful for youth, but such conversations can help youth learn how to cope with ethnic-racial discrimination in the long term. Following a review of studies supporting our conceptual framework, we provide suggestions for future research to expand the field’s understanding of resilience linked to ethnic-racial socialization.

## 1 | INTRODUCTION

Parents of color leverage ethnic-racial socialization to prepare their youth to understand and anticipate aspects about ethnicity-race in their daily lives. *Ethnic-racial socialization* is defined as the behaviors, practices, and social regularities that communicate information about ethnicity-race to youth (Hughes, Harding, et al., 2017).^1^ According to a Gallup (2023) poll, 92% and 83% of African American and Latinx adults, respectively, agreed that youth should learn about African Americans’ contributions to the United States and their impact on society today, and 88% and 84% of African American and Latinx adults agreed that youth should learn about the history of racism in the United States. Similarly, in a Pew (2019) national survey, 64% of African American, 43% of Asian American, and 35% of Latinx adults said that, when they were growing up, their family talked to them often or sometimes about challenges they might face because of their race or ethnicity. Although ethnic-racial socialization is a valuable mechanism through which youth learn about their ethnic-racial groups, the empirical literature has produced mixed results about the effectiveness of ethnic-racial socialization to help youth cope with discrimination. To explain these mixed findings, the present manuscript provides a narrative review of empirical studies examining the protection linked to parents’ ethnic-racial socialization, offers a new conceptual framework drawing from existing theories of resilience, and reviews emergent research supporting our theory of change.

Ethnic-racial socialization is a means to address ethnic-racial discrimination and its consequences. Interpersonal discrimination, as a behavioral component of racism, contributes to disparities in resources, opportunities, and power between ethnic-racial groups (National Research Council, 2004). In a sample of 890 Latinx, Black, Native American, and multi-racial youth, 52%-to-56% of them reported at least one instance of ethnic-racial discrimination from adults in the past year, such as being treated disrespectfully by a store clerk or being hassled by the police because of their ethnicity-race (Kornienko et al., 2023). In a separate study of 1481 Latinx youth, 56%-to-69% of them reported at least one discrimination experience, such as having encountered unfair discipline and receiving low academic expectations from teachers because of their ethnicity-race (Zeiders et al., 2021). During the first few months of the COVID-19 pandemic when anti-Asian hate was especially salient, a survey of 543 Chinese American families found that three out of four Chinese American parents and youth reported at least one incident of vicarious or direct ethnic-racial discrimination (Cheah et al., 2020). In turn, ethnic-racial discrimination is source of stress that is associated with adverse markers of health and well-being among adolescents of color (Del Toro et al., 2021; Del Toro & Hughes, 2019).

To foster ethnic-racial identity development and prepare youth for discrimination, parents of color leverage particular types of ethnic-racial socialization messages. Hughes, Rodriguez et al. (2006) identified four distinct and inter-related ethnic-racial socialization categories: (a) *cultural socialization* are messages that promote ethnic-racial pride, traditions, and history; (b) *preparation for bias* are messages that equip youth with strategies on how to respond to discrimination; (c) *egalitarian* messages acknowledge ethnic-racial diversity yet de-emphasize differences between groups; and (d) *promotion of mistrust* messages encourage caution toward other groups. These dimensions are not mutually exclusive, as cultural socialization can spotlight elders’ perseverance in the struggle for equality and how congruent ethnic-racial figures were instrumental in the struggle for equality (Hughes et al., 2008). Out of the four ethnic-racial socialization types, cultural socialization and preparation for bias are the most commonly studied in relation to discrimination (Umaña-Taylor & Hill, 2020; Wang, Henry, et al., 2019 Wang, Smith, et al., 2019), more favorable). For this reason, from here on, we focus on parental cultural socialization and preparation for bias.

Despite parents’ use of cultural socialization to facilitate youth’s belongingness with their group’s lineage and preparation for bias to help youth adapt in the face of adversity, the role of these practices in protecting adolescents against the effects of discrimination is ambiguous. Some studies found that cultural socialization and preparation for bias reduced the impacts of discrimination (Burt et al., 2017; Neblett et al., 2008); others found that these ethnic-racial socialization messages exacerbated the harms linked to discrimination (Cheeks et al., 2020; Espinoza et al., 2016); still other studies found these ethnic-racial socialization practices did not statistically moderate the consequences of discrimination (Burt & Simons, 2015; Huynh & Fuligni, 2010). These mixed results are perplexing as youth who feel good about and connected to their ethnic-racial group show coping and adaptation in the face of discrimination (Umaña-Taylor, 2024; Yip et al., 2019). Since parents are important sources of information from where adolescents learn to develop such connections with their ethnic-racial groups (Huguley et al., 2019), then the resilience linked to parental cultural socialization and preparation for bias may be manifested in other ways. We propose that cultural socialization and preparation for bias may yield positive responses that are hidden in biological indicators of adolescents’ well-being.

We present a conceptual framework to broaden the field’s understanding, measurement, and interpretation of adolescents’ resilience following ethnic-racial discrimination and parental ethnic-racial socialization. We propose a *Hidden Resilience* framework, wherein resilience among youth may not be immediately apparent in their behaviors and emotions (as measured by psychosocial measures). Rather resilience promoted by ethnic-racial socialization is hidden and evident “under the skin” in long-term regulation and health. To support our Hidden Resilience framework, we provide a narrative review of empirical studies that have tested the moderating roles of parents’ cultural socialization and preparation for bias to illustrate how certain results warrant a re-framing in line with our theory. Then, we draw from extant theories to define and identify Hidden Resilience’s mechanistic properties. We conclude with empirical examples supporting our framework and directions for future research.

## 2 | PARENTAL ETHNIC-RACIAL SOCIALIZATION AS A SOURCE OF ADOLESCENT RESILIENCE

The *Integrative Model for the Study of Developmental Competencies of Minority Children* (García Coll et al., 1996) outlines the relevance of parents’ ethnic-racial socialization. In this framework, several eco-cultural impeding factors, including racism and discrimination, inhibit youth’s developmental competencies. In response to inhibitory societal facets, parents mobilize resources, including community support and cultural specific parenting practices to ensure that their children develop and preserve a positive sense of regard toward their ethnic-racial group (García Coll et al., 1996). African American and Dominican adolescents have been found to be more likely than White adolescents to attribute positive traits to their ethnicity-race (Hughes, Del Toro, et al., 2017), and these positive views have been found to be reliably protective for youth experiencing discrimination (Yip et al., 2019).

### 2.1 | Parental cultural socialization and preparation for bias and adolescent development

Cultural socialization is unequivocally the most beneficial for adolescents (Hughes, 2023). These benefits are attributable to various mechanisms, including elevations in self-esteem, ethnic-racial identity formation, and positive evaluations towards families’ ethnic-racial groups (Reynolds & Gonzales-Backen, 2017). In one review of 259 studies, 61%-to-67% of studies found that cultural socialization was related with positive developmental outcomes (Umaña-Taylor & Hill, 2020). Recent meta-analyses found that cultural socialization was related with higher academic performance, motivation, and engagement (Wang, Smith, et al., 2019), more favorable self-perceptions, interpersonal relationships, and externalizing behaviors (Wang, Henry, et al., 2019), and multiple dimensions of adolescents’ ethnic-racial identities (Huguley et al., 2019).

Relative to cultural socialization, preparation for bias is less clear cut. For instance, in a meta-analysis of 37 studies examining the relations between ethnic-racial socialization and adolescents’ academic outcomes (Wang, Smith, et al., 2019), preparation for bias was, on average, predictive of favorable academic performance, academic motivation, and school engagement. However, turning to a meta-analysis of 102 studies that examined relations between ethnic-racial socialization and adolescents’ psycho-social outcomes (Wang, Henry, et al., 2019), preparation for bias was, on average, linked to more internalizing symptoms, greater externalizing behaviors, but more favorable interpersonal relationships. These divergent results regarding preparation for bias are likely attributable to measurement, as most questionnaires involve youth’s socialization to the existence of racism (Wang, Henry, et al., 2019). In one of the most used measures of preparation for bias (Hughes & Johnson, 2001), parents reported whether they talked to children about racial bias and encouraged youth to work harder than their peers to make up for potential set-backs linked to racism. Because preparation for bias measures include items about discrimination, it can be unclear whether relations between preparation for bias and adolescent development are tainted by unmeasured or unadjusted discrimination experiences.

The mixed results linked to preparation for bias have also been attributable to context (Simon, 2021; Sladek et al., 2022; Umaña-Taylor & Hill, 2020). Preparation for bias includes multiple types of responses to discrimination, including defensive disengagement (e.g., *Do what they tell you and be polite*.), active avoidance (e.g., *Don’t look suspicious and they might leave you alone*.), reflective engagement (e.g., *You know you’*re *not a thief so go about your business and pay them no mind*.), and assertive defiance (e.g., *Stand up for yourself and don’t take any harassment from them*.; Scott et al., 2019). The effectiveness of each type of coping response may depend on context (Smith-Bynum et al., 2016). Among peers who value social status and popularity (Brown, 2011), assertive defiance may be more helpful than defensive engagement for youth to assert and protect themselves from future peer-perpetuated harassment. Conversely, to avoid escalations with teachers and law enforcement that can be detrimental for youth (Bernard et al., 2024; Del Toro, Wang, et al., 2022), families may emphasize more defensive engagement, active avoidance, and reflective engagement than assertive defiance coping responses. Building on extant explanations for the mixed results linked to preparation for bias, we offer an additional framework to understand ethnic-racial socialization in relation to discrimination.

### 2.2 | Parental cultural socialization and preparation for bias vis-à-vis discrimination

**FIGURE 1:**
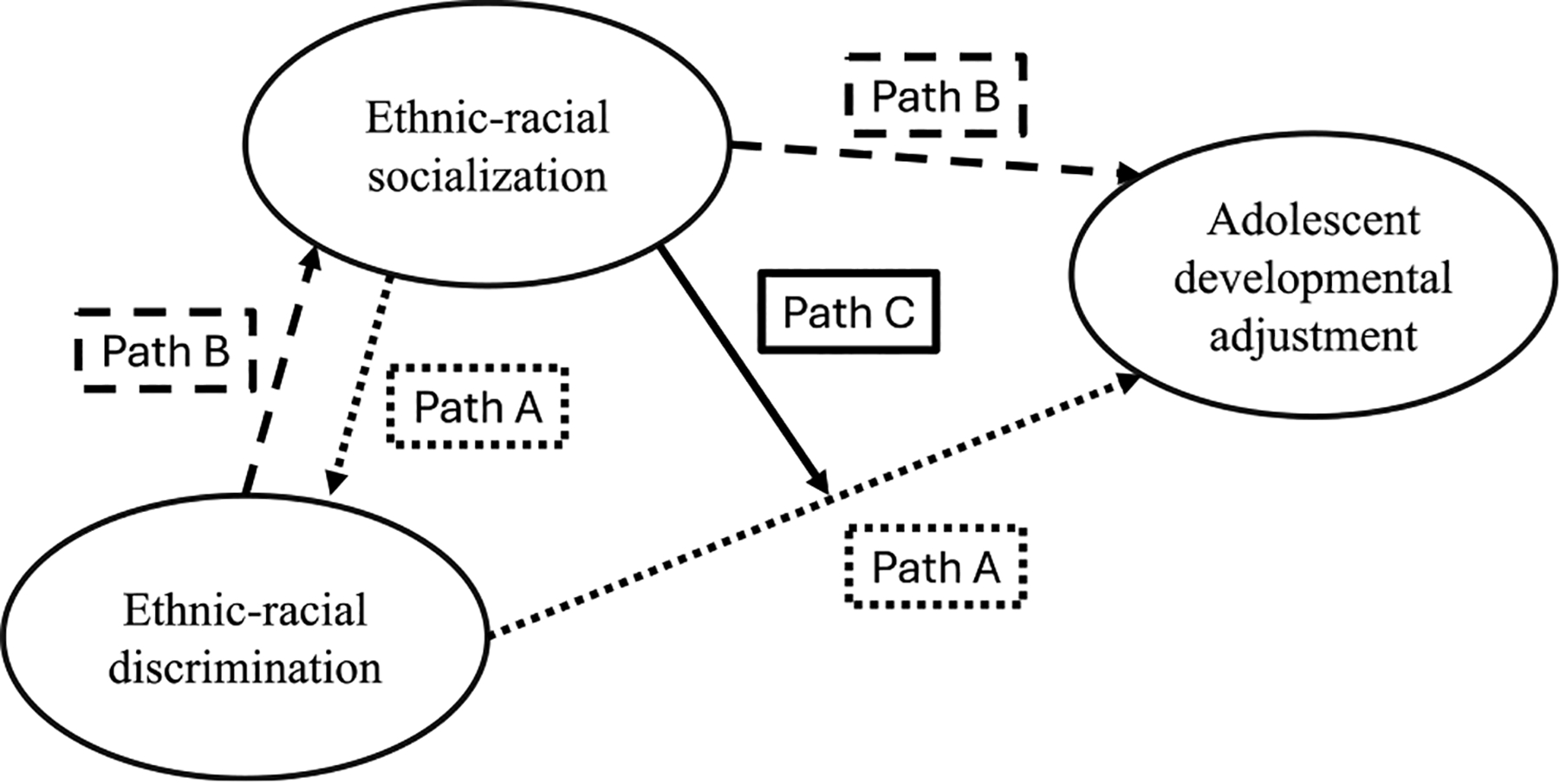
A visual representation of the associations among ethnic-racial discrimination, ethnic-racial socialization, and adolescent developmental adjustment.

Ethnic-racial socialization’s inter-relations with discrimination are dynamic. In one pathway (see Path A in Figure 1), parents’ cultural socialization and preparation for bias can increase youth’s awareness to discrimination and make them readily able to attribute unfair treatment to discrimination, which, in turn, can negatively affect adolescent development (Hughes et al., 2010). In another pathway (see Path B in Figure 1), parents ushering cultural socialization and preparation for bias following discrimination may, in turn, increase their children’s in-group views and distrust in other groups, which can lead youth to self-isolate and show worse adjustment outcomes (Stevenson & Arrington, 2009). For the present manuscript, we focus on the moderating aspects of ethnic-racial socialization or, synonymously, the degree to which ethnic-racial socialization affects the direction or strength of the relation between discrimination and adolescent development (see Path C in Figure 1).

Using Path C in Figure 1 as a guide for our narrative review, we identified 31 empirical studies that tested the moderating aspects of parental ethnic-racial socialization. In line with theory, 23 and 19 studies tested the protective properties of cultural socialization and preparation for bias, respectively. Promotion of mistrust, unspecified types of ethnic-racial socialization, and other types of socialization practices (e.g., egalitarianism) were the least studied, as five, two, and two empirical studies have tested their protective roles, respectively. In addition, all studies in our review relied on psycho-social indicators of adolescents’ adjustment. Specifically, studies examined youth’s behaviors and emotions (*n*-studies = 16), academics (*n*-studies = 8), aspects related to sense of self and identity (*n*-studies = 9), interpersonal and relationship qualities (*n*-studies = 3), but none of these studies included biological markers of stress or health. This focus on psycho-social measures among studies may be partly attributable to behaviors and emotions being easier to collect and measure than biological data. Parents’ ethnic-racial socialization is also conceptualized as natural psycho-social interventions (Del Toro, Karbeah, et al., 2023), positioning psycho-social outcomes as likely outcomes of interest. Thus, our narrative review of parents’ ethnic-racial socialization and adolescents’ discrimination illustrates a gap with an omission of biomarkers of stress and health.

In our review, cultural socialization generally attenuated the adverse effects of discrimination more often than preparation for bias. That is, among the 23 studies that included cultural socialization as a moderator, 52% of them found that cultural socialization reduced the impacts of discrimination. For example, in a three-to-four-year study of Black youth, cultural socialization mitigated the relation between discrimination and conduct problems (Kwon et al., 2022). Similarly, 4-year longitudinal evidence showed that high levels of cultural socialization messages weakened the link between discrimination and depressive symptoms among US-born Filipino American youth (Park et al., 2021). Nonetheless, given that cultural socialization exacerbated or did nothing to statistically moderate the impact of discrimination 48% of the time in existing studies (e.g., Cheeks et al., 2020; Chen et al., 2019; Del Toro & Wang, 2022), the psychological effectiveness of cultural socialization comes with questions.

Relative to cultural socialization, the protective aspects of preparation for bias are more mixed. Among the 19 studies that included preparation for bias, 44% of them found that preparation for bias did not statistically moderate the impact of discrimination (e.g., Atkin et al., 2019; Wang & Huguley, 2012); 25% found that preparation for bias weakened the link between discrimination and youth’s adjustment (e.g., Banerjee et al., 2018; Bynum et al., 2007); 25% found that preparation for bias exacerbated the effect of discrimination on youth’s adjustment (e.g., Saleem et al., 2022; Toomey et al., 2018); and 6% found that preparation for bias was protective for one outcome but problematic for another (e.g., Major, 2020). While scholars put forth several explanations to such discrepancies, including measurement (e.g., awareness vs. coping, Hughes, 2023; Wang, Henry, et al., 2019), content (e.g., defensive vs. assertive responses to discrimination, Scott et al., 2019), and frequency (e.g., possible curvilinear effects; Hughes, Harding, et al., 2017; Seol et al., 2016), the inconsistencies linked to preparation for bias may also be partly attributable to the outcome of interest (e.g., psycho-social measures).

Youth’s psycho-social responses to both ethnic-racial socialization and discrimination are only one piece of multiple levels of outcomes. Following the psychosocial and biological stress that discrimination elicits (Sladek et al., 2021), youth may reach out to their communities to cope with stress, and communities may recognize visible signs of youth’s stress and provide more support to cope with stress (i.e., *Resource Management Theory*; Nguyen et al., 2018; Wheaton, 1985). Youth’s agency following discomfort may enable them to seek out more ethnic-racial socialization, and parents may mobilize more support when they become cognizant of youth’s experiences. For instance, a link between discrimination and psychological distress was strongest when adolescents received high levels of preparation for bias messages in a cross-sectional study of Asian American adolescents (Atkin et al., 2019), and African American youth who received cultural socialization one day reported more negative affect when discrimination happened the following day (Cheeks et al., 2020). However, is ethnic-racial socialization exacerbating the effects of discrimination, or are youth who feel the most hurt by discrimination also seeking out more ethnic-racial socialization? Because most studies rely on psycho-social indicators of adolescent development, the story of resilience linked to ethnic-racial socialization is incomplete.

## 3 | HIDDEN RESILIENCE

Resilience has been studied across multiple systems of functioning and is defined as the capacity of a dynamic system to adapt to challenges that threaten the system’s function, survival, or development (Masten, 2024). *Cellular resilience*, among birds and mammals, highlights how somatic stressors incurred in inflammation is important in the development of longevity, with longer-lived species showing greater resilience to immune-related stressors (Finch et al., 2010). *Biological resilience*, largely studied in mice, is one’s innate biological abilities to recover after deviating from damage caused by stress (Kirkland et al., 2016; Ukraintseva et al., 2021). Among humans, *organ resilience* refers to how organs, such as one’s cardiovascular system, responds to inflammation and diseases (Logan & Barksdale, 2008). Therefore, multiple systems (e.g., genes, biology, and contexts) interact with one another to help individuals’ cope and adapt in times of adversity in ways that may not be visible to the naked eye. We leverage these theories to propose a testable framework wherein adolescents’ biological resilience following ethnic-racial socialization may be an outcome of momentary and benign psycho-social discomfort.

### 3.1 | Existing models as origins of the hidden resilience model

The idea that there might be a trade-off between psycho-social stress in exchange for long-term physical health comes from the following two theories: *The Environment Affordances Model* (Mezuk et al., 2013) and *Skin-Deep Resilience* (Brody et al., 2013). The Environment Affordances Model describes the contextual underpinnings shaping the mental-physical health paradox, wherein African Americans report favorable mental health but show worse physical health relative to White Americans (Jackson & Knight, 2006; Mezuk et al., 2013). Due to segregation and consequences associated with red lining, African Americans live in more socioeconomically disadvantaged neighborhoods and White Americans live in more socioeconomically affluent neighborhoods, and these neighborhoods constitute disparities in resources and in opportunities for these two social groups to be resilient. For instance, in neighborhoods that are predominantly African American, more health-impeding resources, such as fast-food chains and liquor stores, may provide comfort in the short-term but are detriments to one’s long-term physical health. Conversely, in neighborhoods that are predominantly White, more health-promoting resources (e.g., salad-restaurants, quality gyms) are available that may not feel good in the short term but are beneficial to one’s physical health in the long term.

Turning next to Skin-Deep Resilience, individuals who strive and achieve upward social mobility show worse physical health (Chen et al., 2022). Skin-Deep Resilience is premised on *John Henryism Theory*, which tells the story of John Henry and his determination to succeed and harness his strength to overpower a mechanical drill. Unfortunately, to best the mechanical drill, Henry engaged in high-effort coping and chronic sympathetic nervous system arousal, which deteriorated Henry’s health from exhaustion (Brody et al., 2018; James, 1994). In line with Henry’s story, studies show that youth of color who persevere in school and work hard to overcome adversities pay costly tolls with their physical well-being, partly attributable to their increased exposure to White spaces (Chen et al., 2022). This inter-group contact are potential sources of lack of belonging and mismatches between under-represented individuals’ cultural norms and the environments’ norms predominantly serving White Americans (Chen et al., 2022).

According to the Environmental Affordances Model and Skin-Deep Resilience, psycho-social and biological measures tell opposing stories. Specifically, at the cost of striving to feel good and happy in life, individuals report favorable psycho-social outcomes but show worse biomarkers of health. Rather than understanding the consequences associated with risk factors (e.g., segregation), we theorize an inverse pathway that may be possible and linked to individuals’ engagement in effective resilient practices. Specifically, discriminated youth who report more psychosocial distress may be aware of the impacts tied to discrimination and may have preemptively developed adaptive short-term psychological and biological stress responses and engage in routine health behaviors and cognitive re-appraisals, which would enable such youth to show better biological indicators of health. One means through which youth may be learning about these practices and expanding their purviews to cope with discrimination is parental ethnic-racial socialization. In our model, the process through which discriminated youth show resilience in biological markers of well-being and healthy aging following ethnic-racial socialization is deemed hidden resilience.

### 3.2 | Overview of the hidden resilience model

**FIGURE 2:**
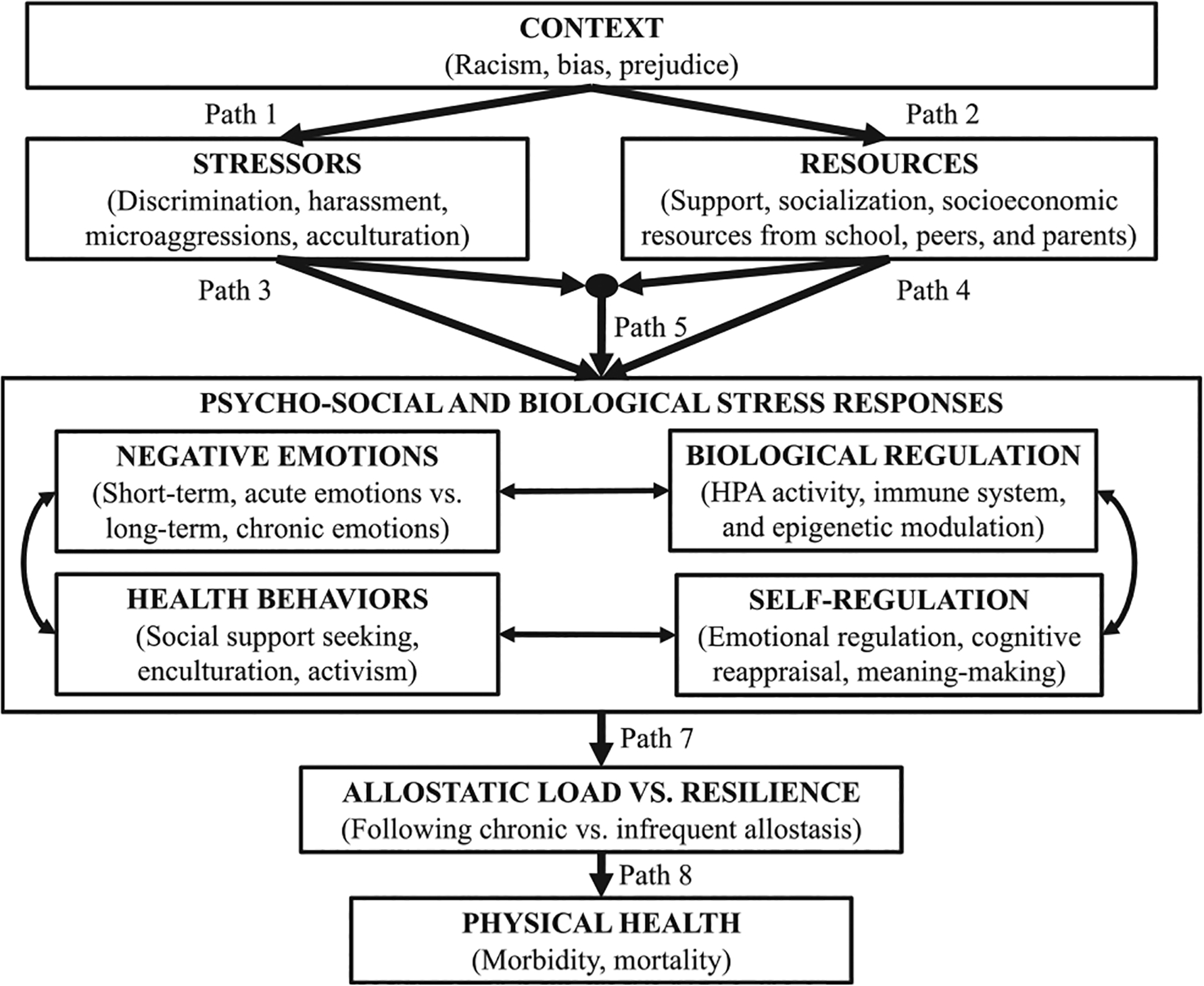
The hidden resilience model.

Figure 2 outlines our Hidden Resilience Model. In line with the dynamic-systems component of resilience (Masten, 2024), Figure 2 illustrates the multilevel and inter-connected nature of stressors and resources contributing to adolescents’ hidden resilience. Racism is a historical and structural feature that shapes daily experiences among individuals, including micro-aggressions and discrimination. Adolescents also are surrounded by multiple socializing agents, such as peers, educators, and parents (Sladek et al., 2022), that may proactively and reactively respond to racism and its interpersonal properties (e.g., microaggressions) via social support (Brody et al., 2016) and ethnic-racial socialization (Del Toro & Wang, 2021b; Nelson et al., 2018). In addition, schools, peers, and parents have facets (e.g., identities, socioeconomic resources) that can contribute to children’s resilience through norms, social regularities, and physical-architectural features (Barton et al., 2019; Brody et al., 2019; Del Toro & Wang, 2021a).

For simplicity, discrimination and ethnic-racial socialization are the foci of the present manuscript’s stressors and resources. Hidden Resilience rests on three premises: (1) The efficacy of ethnic-racial socialization to promote adolescents’ well-being could not be well understood without accounting for adolescents’ discrimination; (2) to understand whether ethnic-racial socialization instills youth with resilience against discrimination, an assessment of how discrimination and ethnic-racial socialization interact with one another to predict adolescents’ developmental outcomes is a foundational step; and (3) youth’s resilience against discrimination may not be noticeable in psycho-social measures but is hidden underneath the skin. Biomarkers may capture youth’s *routine* and *consistent* engagement in practices (e.g., exposure to short-term stressors, health behaviors, and cognitive re-appraisals) that can offer them protection against discrimination over time. For instance, the initial stages of an exercise or a healthy diet may not immediately feel good nor generate significant change, but a routine exercise and a consistent healthy diet are advantageous for one’s physical health. Because these practices need to be routine, they may not offer protection that is immediately apparent in biological measures of healthy human functioning.

In our Hidden Resilience model, discrimination as a fruition of racism influences adolescent well-being (see Paths 1 and 3). Feeling threatened and a lack of control following discrimination can activate adolescents’ primary biological stress mediators, such as the sympathetic-adrenal-medullary (SAM) system and the hypothalamic-pituitary-adrenal (HPA) axis (Adam et al., 2015; Dickerson & Kemeny, 2004). Routine negative evaluations from others derived from discrimination can elicit short-term primary biological stress responses (part of an adaptive regulatory process known as allostasis; McEwen & Seeman, 1999). However, recall that chronic elevations in biological stress from repeated discrimination experiences can lead to alterations in primary biological stress systems and the downstream biological systems they regulate (e.g., inflammation), resulting in a multi-system biological dysregulation known as allostatic load (Adam et al., 2015; Dickerson & Kemeny, 2004). As an example, in cortisol as a multi-faceted index of allostatic load among social groups that are chronically exposed to marginalization (Juster et al., 2010), discrimination is, on average, associated with greater total cortisol output in the short term (Gunnar et al., 2022) and lower total cortisol output and flatter diurnal cortisol slopes in the long term (Adam et al., 2015); these dysregulations in cortisol, in turn, are associated with worse health measures, including inflammation and immune system outcomes (Adam et al., 2017).

Controlling for these adverse effects linked to discrimination, parental ethnic-racial socialization can have promotive effects on adolescents’ biological stress responses (see Path 4). Although studies have not tested relations between ethnic-racial socialization and biomarker indicators of adolescent well-being, these relations are possibly favorable. In one study of 103 Mexican-origin adolescents, youth who felt good about their ethnic-racial identities showed steeper declines in diurnal cortisol slopes than did youth who felt less good about their ethnic-racial identities (Zeiders et al., 2017). In a separate study of 50 Black youth, those who had stronger feelings about and connections to one’s ethnic-racial community marginally showed steeper declines in diurnal cortisol slopes relative to their peers who had less favorable views (Adam et al., 2020). Both studies controlled for youth’s self-reported discrimination, ruling out the possibility that these links may have been attributable to youth’s infrequent discrimination experiences. With parents as likely sources of youth’s ethnic-racial identities (Huguley et al., 2019), parental ethnic-racial socialization may directly be related to positive biomarker indicators of adolescent health.

In addition to its direct effects on adolescent development, parental ethnic-racial socialization may interact with discrimination to predict youth’s resilience against the impacts linked to discrimination (see Path 5). One mechanism we put forth is via psycho-social and biological stress responses. The psycho-social responses highlighted in our framework (e.g., negative emotions) are momentary and benign. Relative to chronic psycho-social stressors that last between weeks, months, and years, short-term psycho-social stressors last between minutes and days. These differences in duration between stressors are partly attributable their contrasting triggers, as momentary stressors come from acute events (e.g., receiving feedback) whereas those that are chronic come from periodic circumstances (e.g., discrimination). In turn, momentary stress responses include increased heart rate variability, heightened alertness, and stress hormonal releases, but chronic psycho-social stress responses include prolonged activation of biological stress response systems, sustained stress hormonal elevations, and dysregulated physiological processes (Adam et al., 2023). In turn, short-term stress—although momentarily unpleasant—is generally manageable and can enhance performance and motivation in certain situations (Dhabhar, 2018), versus chronic stress is linked to increased cardiovascular disease, weakened immune function, and mood disorders like depression and anxiety (Adam et al., 2023). In line with the distinction between short-versus long-term psycho-social stressors, ethnic-racial socialization may generate benign discomfort that can enhance youth’s resilience.

In line with our theory, cultural socialization and preparation for bias have been found to generate momentary discomfort. For instance, both parents and children were found to report some discomfort when engaging in preparation for bias (Hughes, Bachman, et al., 2006). In addition, across three daily diaries among African American, Latinx, and Asian American adolescents (Cheeks et al., 2020; Del Toro, Atkin, et al., accepted), on days when youth reported more cultural socialization than their own average reported more—although non-significant—negative emotions that same day. These benign upticks in short-term stress responses may provide youth with energetic and attentional resources for coping with stress, and preemptively prepare immune cell distribution, function, and primary/secondary responses to be adaptive and responsive to stressors that may be chronic (Dhabhar, 2018).

An additional means adolescents develop resilience against discrimination is via their health behaviors. In line with extant frameworks that position health behaviors as an impetus for trade-offs between psycho-social and biological measures (Jackson & Knight, 2006), we theorize that routine self-care activities following ethnic-racial socialization and discrimination can lead to more favorable health outcomes. In one of the largest studies examining risk factors linked to cardiovascular diseases among Black and White adults aged 18-to-30 (i.e., the Coronary Artery Risk Development in Young Adults, CARDIA, study; Borrell et al., 2013), African Americans experiencing moderate or high levels of discrimination were more physically active than those reporting no discrimination. While it may be possible that individuals who spend more time outdoors and public spaces (e.g., gyms) are more likely to have social interactions with others, including discrimination encounters, a randomized controlled trial leveraging computerized interactive patient education among 930 patients across 15 clinics found that discrimination was associated with worse dieting at baseline, but the 12-month trial improved the healthy eating behaviors among adults experiencing the most frequent discrimination encounters (Forsyth et al., 2014). Therefore, regardless of outcome (e.g., exercise, diet), individuals who are more exposed to discrimination may utilize more resources available to them to cope with discrimination.

Ethnic-racial socialization may also instill youth with resilience against discrimination via alterations in adolescent cognitive processing. Following discrimination, youth can feel a lack of control and internalize negative stereotypes (Dickerson & Kemeny, 2004). This internalization can be interrupted via cultural socialization and preparation for bias. Cultural socialization, including messages about how same-race figures contributed to the struggle for equality (Hughes et al., 2008), can frame youth’s discrimination perceptions as surmountable. Preparation for bias can prepare youth to attribute discrimination to institutional prejudices as opposed to individual-level qualities (e.g., victim blaming; Major et al., 2003), and youth who learn to make these attributions may not undergo rumination and internalization of negative worldviews following discrimination. These altered cognitive interpretations of stress may, in turn, weaken the negative relationship between discrimination and health.

Indeed, parental ethnic-racial socialization has been found to predict youth’s beliefs that can shape their health. In one study of 367 adolescents of color (Christophe et al., 2024), youth who received more cultural socialization and preparation for bias, on average, reported more frequent adjustments to their responses to stress via emotion regulation and persistence following adversity (i.e., *Shift-and-Persist*; Chen & Miller, 2012). In a separate but related literature, youth who shift-and-persist were found to show low levels of allostatic load, including lower inflammation and obesity, following adversity (for a review, see Chen et al., 2024). Although these separate literatures support our framework in a piece-wise fashion, shift-and-persist may be a mechanism through which ethnic-racial socialization leads to biological resilience among discriminated youth.

### 3.3 | Existing studies supporting hidden resilience

In two recent studies incorporating adolescents’ biomarkers, youth show evidence of hidden resilience. For instance, in one study using longitudinal data from the Adolescent Brain Cognitive Development study, Del Toro, Anderson, et al. (accepted) examined relations among adolescents’ discrimination, pubertal development, and their parents’ ethnic-racial identities. Pubertal development that takes place more advanced relative to one’s same-age and same-sex peers can be a consequence of accelerated aging due to stress (Deardorff et al., 2019), which has evolutionary underpinnings wherein chronic threats to the human body can trigger early pubertal onset to ensure one’s survival and secure continuity of one’s lineage (Gluckman & Hanson, 2006). Discrimination is a source of stress that contributes to more advanced pubertal development among adolescents of color relative to their White peers (Deardorff et al., 2019). Parents’ beliefs and practices about how to protect their youth from the harms linked to discrimination may come from their ethnic-racial identities. In particular, parents’ ethnic-racial identity commitment, which is a sense of belonging and connection with an ethnic-racial group (Phinney & Ong, 2007), may offer the most protection because the nature of identity as a consistent sense of self across time and context (Erikson, 1994). Parents with high ethnic-racial identity commitment may routinely expose their youth to others with similar ethnic-racial identities, and this chronic exposure to their communities may confer discriminated youth with protection as such youth may be more attuned to biased treatment and visual representations of their ethnic-racial group. In addition, parents who feel a strong sense of connection to their group have reported more frequent cultural socialization and preparation for bias practices (Holloway & Varner, 2021; Hughes & Johnson, 2001), which could be mediators between parents’ identities and their youth’s resilience. Indeed, above and beyond age-differences, adolescents who experienced more discrimination than their siblings showed more advanced pubertal development, and this within-sibling effect was weakened for siblings in households wherein parents reported high levels of ethnic-racial identity commitment (Del Toro, Anderson, et al., accepted). Notably, discrimination predicted more advanced pubertal development 1 year later, but pubertal development did not predict more discrimination 1 year later, ruling out the possibility that youth in the study who physically matured earlier than their peers solicited more prejudicial treatment from unfamiliar strangers (e.g., Goff et al., 2014).

In a second study using data from the Future of Families and Child Wellbeing study, Del Toro, Karbeah, et al. (2023) examined relations among youth’s police intrusion, epigenetic age acceleration (EAA), and their fathers’ ethnic-racial identity commitment. Recent epigenetic clocks in the literature were derived to estimate EAA, which can be a molecular index of advanced biological aging due to stress (Raffington & Belsky, 2022). On average, youth of color who experienced more police intrusion (e.g., racial slurs) by age 13 were more likely to show more EAA at age 15, above and beyond age-9 EAA. In addition, fathers’ ethnic-racial identity commitment weakened the relation between youth’s police intrusion and EAA. The protection linked to fathers’ ethnic-racial identity commitment may be attributable to fathers having prior legal system exposure (Del Toro, Fine, et al., 2022), which may be a source of knowledge about how one can preserve one’s ethnic-racial identity in the face of surveillance that associates one’s ethnicity-race with negative stereotypes, including criminality (Goff et al., 2014). Although both studies focused on parents’ ethnic-racial identities, parents’ ethnic-racial socialization may have mediated the link between their ethnic-racial identities and adolescents’ biological resilience.

## 4 | IMPLICATIONS AND CONCLUSION

Integrating biology into the study of ethnic-racial socialization may enhance the depiction of resilience among communities of color. Because there is a wide array of markers of biological processes with diverse implications for human functioning (e.g., diurnal cortisol, inflammation; Adam & Kumari, 2009), incorporating indicators of human biology can augment the field’s understanding about how discriminated adolescents respond to parental ethnic-racial socialization. In line with extant theory describing the diverse ecological levels and their contributions to children’s development (i.e., *Bio-Ecological Model of Human Development*; Bronfenbrenner & Morris, 2006), scholars should study the *bio* component more often to have a robust apparatus of development among children of color experiencing discrimination.

The goal of the present paper was to propose a framework amplifying families’ resilience in the context of ethnic-racial discrimination. Our focus on families should not detract from the fact that policies need to minimize discrimination in communities of color. Scholars often leave families with the burden to develop resilience within a stratified society, without directly changing the policies and practices that perpetuate racism and discrimination (Chae et al., 2021). Our innovative framework, *Hidden Resilience*, integrates social surveys and biological data to demonstrate that adolescents may embody their families’ engagement in resilience-promoting behaviors and regularities, including ethnic-racial socialization. While interventions are necessary to promote youth resilience, another focus should be on targeting settings and policies that give rise to discrimination toward adolescents of color. Because there are multiple policies and institutions (e.g., education, medicine, law enforcement) in the United States that may intentionally and unintentionally perpetuate discrimination, we welcome future work to specify their setting level intervention. In line with extant propositions (Del Toro, Jackson et al., 2023; Sullivan & O’Keeffe, 2017), curtailing policies across multiple institutions that perpetuate discrimination will improve the public health of America’s children.

## Data Availability

Data sharing not applicable to this article as no datasets were generated or analyzed during the current study.

## Footnotes

1 In the literature, there are various terms for ethnic socialization, racial socialization, and ethnic-racial socialization. In line with Hughes, Rodriguez et al. (2006), we use the combined term *ethnic-racial socialization* for the sake of brevity and to avoid further fragmenting the literature when classifications of groups as *ethnic* versus *racial* change over time.

## References

[R1] Adam EK, Collier Villaume S, Thomas S, Doane LD, & Grant KE (2023). Stress and hypothalamic–pituitary–adrenal axis activity in adolescence and early adulthood. In APA handbook of adolescent and young adult development (pp. 55–72). American Psychological Association. 10.1037/0000298-004

[R2] Adam EK, Heissel JA, Zeiders KH, Richeson JA, Ross EC, Ehrlich KB, Levy DJ, Kemeny M, Brodish AB, Malanchuk O, Peck SC, Fuller-Rowell TE, & Eccles JS (2015). Developmental histories of perceived racial discrimination and diurnal cortisol profiles in adulthood: A 20-year prospective study. Psychoneuroendocrinology, 62, 279–291. 10.1016/j.psyneuen.2015.08.018 26352481 PMC4739843

[R3] Adam EK, Hittner EF, Thomas SE, Villaume SC, & Nwafor EE (2020). Racial discrimination and ethnic racial identity in adolescence as modulators of HPA axis activity. Development and Psychopathology, 32(5), 1669–1684. Cambridge Core. 10.1017/S095457942000111X 33427170

[R4] Adam EK, & Kumari M (2009). Assessing salivary cortisol in large-scale, epidemiological research. Psychoneuroendocrinology, 34(10), 1423–1436. 10.1016/j.psyneuen.2009.06.011 19647372

[R5] Adam EK, Quinn ME, Tavernier R, McQuillan MT, Dahlke KA, & Gilbert KE (2017). Diurnal cortisol slopes and mental and physical health outcomes: A systematic review and meta-analysis. Psychoneuroendocrinology, 83, 25–41. 10.1016/j.psyneuen.2017.05.018 28578301 PMC5568897

[R6] Atkin AL, Yoo HC, & Yeh CJ (2019). What types of racial messages protect Asian American adolescents from discrimination? A latent interaction model. Journal of Counseling Psychology, 66(2), 247–254. 10.1037/cou0000297 30035592

[R7] Banerjee M, Byrd C, & Rowley S (2018). The relationships of school-based discrimination and ethnic-racial socialization to African American adolescents’ achievement outcomes. Social Sciences, 7(10), 208. Publicly Available Content Database. 10.3390/socsci7100208

[R8] Barton AW, Brody GH, Yu T, Kogan SM, Chen E, & Ehrlich KB (2019). The profundity of the everyday: Family routines in adolescence predict development in young adulthood. Journal of Adolescent Health, 64(3), 340–346. 10.1016/j.jadohealth.2018.08.029 PMC9389627 30392861

[R9] Bernard DL, Saleem FT, Moreland AD, Shacklewood C, & Danielson CK (2024). A qualitative analysis of Black mother preparation for bias messages following incidents of racism-related violence. Journal of Family Psychology, 38(1), 38–47. 10.1037/fam0001162 37917492 PMC10842490

[R10] Borrell LN, Kiefe CI, Diez-Roux AV, Williams DR, & Gordon-Larsen P (2013). Racial discrimination, racial/ethnic segregation, and health behaviors in the CARDIA study. Ethnicity and Health, 18(3), 227–243. 10.1080/13557858.2012.713092 22913715 PMC3523091

[R11] Brody GH, Miller GE, Yu T, Beach SRH, & Chen E (2016). Supportive family environments ameliorate the link between racial discrimination and epigenetic aging: A replication across two longitudinal cohorts. Psychological Science, 27(4), 530–541. 10.1177/0956797615626703 26917213 PMC4833531

[R12] Brody GH, Yu T, Chen E, Miller GE, Kogan SM, & Beach SRH (2013). Is resilience only skin deep? Rural African Americans’ socioeconomic status–related risk and competence in preadolescence and psychological adjustment and allostatic load at age 19. Psychological Science, 24(7), 1285–1293. 10.1177/0956797612471954 23722980 PMC3713113

[R13] Brody GH, Yu T, Miller GE, Ehrlich KB, & Chen E (2018). John Henryism coping and metabolic syndrome among young Black adults. Psychosomatic Medicine, 80(2), 216–221. 10.1097/PSY.0000000000000540 29140885 PMC5794531

[R14] Brody GH, Yu T, Nusslock R, Barton AW, Miller GE, Chen E, Holmes C, McCormick M, & Sweet LH (2019). The protective effects of supportive parenting on the relationship between adolescent poverty and resting-state functional brain connectivity during adulthood. Psychological Science, 30(7), 1040–1049. 10.1177/0956797619847989 31088209 PMC6657149

[R15] Bronfenbrenner U, & Morris PA (2006). The Bioecological model of human development. In Handbook of child psychology: Theoretical models of human development (6th ed., Vol. 1, pp. 793–828). John Wiley & Sons Inc. 10.1002/9780470147658.chpsy0114

[R16] Brown BB (2011). Popularity in peer group perspective: The role of status in adolescent peer systems. In Popularity in the peer system (pp. 165–192). Guilford Press.

[R17] Burt CH, Lei MK, & Simons RL (2017). Racial discrimination, racial socialization, and crime: Understanding mechanisms of resilience. Social Problems, 64(3), 414–438. 10.1093/socpro/spw036 PMC13372252 42459501

[R18] Burt CH, & Simons RL (2015). Interpersonal racial discrimination, ethnic-racial socialization, and offending: Risk and resilience among African American females. Justice Quarterly, 32(3), 532–570. 10.1080/07418825.2013.781205 PMC13268400 42306196

[R19] Bynum MS, Burton ET, & Best C (2007). Racism experiences and psychological functioning in African American college freshmen: Is racial socialization a buffer? Cultural Diversity and Ethnic Minority Psychology, 13(1), 64–71. 10.1037/1099-9809.13.1.64 17227178

[R20] Chae DH, Snipes SA, Chung KW, Martz CD, & LaVeist TA (2021). Vulnerability and resilience: Use and misuse of these terms in the public health discourse. American Journal of Public Health, 111(10), 1736–1740. 10.2105/AJPH.2021.306413 34554819 PMC8561197

[R21] Cheah CSL, Wang C, Ren H, Zong X, Cho HS, & Xue X (2020). COVID-19 racism and mental health in Chinese American families. Pediatrics, 146(5). 10.1542/peds.2020-021816 32873719

[R22] Cheeks BL, Chavous TM, & Sellers RM (2020). A daily examination of African American adolescents’ racial discrimination, parental racial socialization, and psychological affect. Child Development, 91(6), 2123–2140. 10.1111/cdev.13416 32767759

[R23] Chen E, Brody GH, & Miller GE (2022). What are the health consequences of upward mobility? Annual Review of Psychology, 73(1), 599–628. 10.1146/annurev-psych-033020-122814 PMC10142907 34579546

[R24] Chen E, Jiang T, Chen MA, & Miller GE (2024). Reflections on resilience. Development and Psychopathology, 1–8. 10.1017/S0954579424000403 PMC11341778 38389301

[R25] Chen E, & Miller GE (2012). “Shift-and-Persist” strategies: Why low socioeconomic status isn’t always bad for health. Perspectives on Psychological Science, 7(2), 135–158. 10.1177/1745691612436694 23144651 PMC3491986

[R26] Chen S, Benner A, & Wang Y (2019). Discrimination and adolescents’ academic and socioemotional adjustment: The moderating roles of family and peer cultural socialisation. International Journal of Psychology, 55(5), 702–712. n/a(n/a). 10.1002/ijop.12637 31788794 PMC10704380

[R27] Christophe NK, Stein GL, & Salcido VV (2024). Parental ethnic–racial socializations messages direct and indirect associations with shift-and-persist coping among minoritized American adolescents. Cultural Diversity and Ethnic Minority Psychology. 10.1037/cdp0000637 38300600

[R28] Deardorff J, Hoyt LT, Carter R, & Shirtcliff EA (2019). Next steps in puberty research: Broadening the lens toward understudied populations. Journal of Research on Adolescence, 29(1), 133–154. 10.1111/jora.12402 30869847 PMC6827435

[R29] Del Toro J, Anderson RE, Sun X, & Lee R (accepted). Early adolescents’ ethnic-racial discrimination and pubertal development: Parents’ ethnic-racial Identities promote adolescents’ resilience. American Psychologist. 10.1037/amp0001284 PMC11959565 39531710

[R30] Del Toro J, Atkin AL, Golden AR, Ip K, & Wang M-T (accepted). Ethnic/racial discrimination, school cultural socialization, and negative affect: Daily diaries reveal African American, Asian American, and Latinx adolescents’ resilience. Journal of Educational Psychology. 10.1037/edu0000893

[R31] Del Toro J, Fine A, Wang M-T, Thomas A, Schneper LM, Mitchell C, Mincy RB, McLanahan S, & Notterman DA (2022a). The longitudinal associations between paternal incarceration and family well-being: Implications for ethnic/racial disparities in health. Journal of the American Academy of Child & Adolescent Psychiatry, 61(3), 423–433. 10.1016/j.jaac.2021.08.005 34389441 PMC8828798

[R32] Del Toro J, & Hughes D (2019). Trajectories of discrimination across the college years: Associations with academic, psychological, and physical adjustment outcomes. Journal of Youth and Adolescence, 49(4), 772–789. 10.1007/s10964-019-01147-3 31650443

[R33] Del Toro J, Hughes D, & Way N (2021). Inter-relations between ethnic-racial discrimination and ethnic-racial identity among early adolescents. Child Development, 92(1), e106–e125. 10.1111/cdev.13424 32780881

[R34] Del Toro J, Jackson DB, Testa A, & Wang M-T (2023a). The spillover effects of classmates’ police intrusion on adolescents’ school-based defiant behaviors: The mediating role of institutional trust. American Psychologist, 78(8), 941–954. 10.1037/amp0001148 36913279

[R35] Del Toro J, Karbeah J, Jackson DB, & Fine A (2023b). Can adolescents show resilience under the skin? Fathers’ ethnicracial identities protect children against the accelerated epigenetic aging linked to police intrusion. In Yip T (Ed.), Cambridge handbook of ethnic/racial discrimination and youth development. Retrieved from https://osf.io/preprints/psyarxiv/8kwrs/

[R36] Del Toro J, & Wang MT (2021a). Longitudinal inter-relations between school cultural socialization and school engagement: The mediating role of school climate. Learning and Instruction, 75, 101482. 10.1016/j.learninstruc.2021.101482 33442773

[R37] Del Toro J, & Wang M-T (2021b). School cultural socialization and academic performance: Examining ethnic-racial identity development as a mediator among African American Adolescents. Child Development, 92(4), 1458–1475. 10.1111/cdev.13467 33205402

[R38] Del Toro J, & Wang M-T (2022). Police stops and school engagement: Examining cultural socialization from parents and schools as protective factors among African American adolescents. American Educational Research Journal, 60(1), 00028312221132533. 10.3102/00028312221132533

[R39] Del Toro J, Wang M-T, Thomas A, & Hughes D (2022b). An intersectional approach to understanding the academic and health effects of policing among urban adolescents. Journal of Research on Adolescence, 32(1), 34–40. 10.1111/jora.12685 34605113

[R40] Dhabhar FS (2018). The short-term stress response: Mother nature’s mechanism for enhancing protection and performance under conditions of threat, challenge, and opportunity. Frontiers in Neuroendocrinology, 49, 175–192. 10.1016/j.yfrne.2018.03.004 29596867 PMC5964013

[R41] Dickerson SS, & Kemeny ME (2004). Acute stressors and cortisol responses: A theoretical integration and synthesis of laboratory research. Psychological Bulletin, 130(3), 355–391. 10.1037/0033-2909.130.3.355 15122924

[R42] Erikson EH (1994). Identity and the life cycle. WW Norton & company.

[R43] Espinoza G, Gonzales NA, & Fuligni AJ (2016). Parent discrimination predicts Mexican-American adolescent psychological adjustment 1 year later. Child Development, 87(4), 1079–1089. 10.1111/cdev.12521 27224903 PMC4939107

[R44] Finch CE, Morgan TE, Longo VD, & de Magalhaes JP (2010). Cell resilience in species life spans: A link to inflammation? Aging Cell, 9(4), 519–526. 10.1111/j.1474-9726.2010.00578.x 20415721 PMC2952360

[R45] Forsyth JM, Schoenthaler A, Ogedegbe G, & Ravenell J (2014). Perceived racial discrimination and adoption of health behaviors in hypertensive Black Americans: The CAATCH trial. Journal of Health Care for the Poor and Underserved, 25(1), 276–291. 10.1353/hpu.2014.0053 24509026 PMC12516789

[R46] Gallup. (2023). Majorities support racial education in U.S. Schools. Gallup.Com. Retrieved from https://news.gallup.com/poll/508172/majorities-support-racial-education-schools.aspx

[R47] García Coll C, Lamberty G, Jenkins R, McAdoo HP, Crnic K, Wasik BH, & Vazquez Garcia H (1996). An integrative model for the study of developmental competencies in minority children. Child Development, 67(5), 1891–1914. 10.1111/j.1467-8624.1996.tb01834.x 9022222

[R48] Gluckman PD, & Hanson MA (2006). Evolution, development and timing of puberty. Trends in Endocrinology and Metabolism: TEM, 17(1), 7–12. 10.1016/j.tem.2005.11.006 16311040

[R49] Goff PA, Jackson MC, Di Leone BAL, Culotta CM, & DiTomasso NA (2014). The essence of innocence: Consequences of dehumanizing Black children. Journal of Personality and Social Psychology, 106(4), 526–545. 10.1037/a0035663 24564373

[R50] Gunnar MR, Haapala J, French SA, Sherwood NE, Seburg EM, Crain AL, & Kunin-Batson AS (2022). Race/ethnicity and age associations with hair cortisol concentrations among children studied longitudinally from early through middle childhood. Psychoneuroendocrinology, 144, 105892. 10.1016/j.psyneuen.2022.105892 35985241

[R51] Holloway K, & Varner F (2021). Parenting despite discrimination: Does racial identity matter? Cultural Diversity and Ethnic Minority Psychology, 27(4), 781–795. 10.1037/cdp0000452 34279979 PMC8497417

[R52] Hughes D (2023). Research and scholarship on racial socialization: Getting here. In Witherspoon DP, McHale SM, & King V (Eds.), Family socialization, race, and inequality in the United States (pp. 63–92). Springer Nature. 10.1007/978-3-031-44115-8_4

[R53] Hughes D, Bachman MA, Ruble DN, & Fuligni A (2006a). Tuned in or tuned out: Parents’ and children’s interpretation of parental racial/ethnic socialization practices. In Balter L & Tamis-LeMonda C (Eds.), Child psychology: A handbook of contemporary issues (2nd ed., pp. 591–610). Psychology Press.

[R54] Hughes D, Del Toro J, & Way N (2017a). Interrelations among dimensions of ethnic-racial identity during adolescence. Developmental Psychology, 53(11), 2139–2153. 10.1037/dev0000401 29094976

[R55] Hughes D, Hagelskamp C, Del Toro J, Shrout P, & Way N (2010). Reciprocity in longitudinal relations between ethnicracial socialization, discrimination, and ethnic-identity: Autoregressive latent trajectory analysis.

[R56] Hughes D, Harding JF, Niwa EY, Del Toro J, & Way N (2017b). Racial socialization and racial discrimination as intraand inter-group processes. In Rutland A, Nesdale D, & Brown CS (Eds.), The Wiley-Blackwell handbook of group processes in children adolescents (pp. 243–268). Wiley.

[R57] Hughes D, & Johnson D (2001). Correlates in children’s experiences of parents’ racial socialization behaviors. Journal of Marriage and Family, 63(4), 981–995. 10.1111/j.1741-3737.2001.00981.x

[R58] Hughes D, Rivas D, Foust M, Hagelskamp C, Gersick S, & Way N (2008). How to catch a moonbeam: A mixed-methods approach to understanding ethnic socialization processes in ethnically diverse families. In Quintana SM & McKown C (Eds.), Handbook of race, racism, and the developing child (pp. 226–277). John Wiley & Sons Inc.

[R59] Hughes D, Rodriguez J, Smith EP, Johnson DJ, Stevenson HC, & Spicer P (2006b). Parents’ ethnic-racial socialization practices: A review of research and directions for future study. Developmental Psychology, 42(5), 747–770. 10.1037/0012-1649.42.5.747 16953684

[R60] Huguley JP, Wang M-T, Vasquez AC, & Guo J (2019). Parental ethnic–racial socialization practices and the construction of children of color’s ethnic–racial identity: A research synthesis and meta-analysis. Psychological Bulletin, 145(5), 437–458. 10.1037/bul0000187 30896188

[R61] Huynh VW, & Fuligni AJ (2010). Discrimination hurts: The academic, psychological, and physical well-being of adolescents. Journal of Research on Adolescence, 20(4), 916–941. 10.1111/j.1532-7795.2010.00670.x

[R62] Jackson JS, & Knight KM (2006). Race and self-regulatory health behaviors: The role of the stress response and the HPA axis in physical and mental health disparities. In Social structures, aging, and self-regulation in the elderly (pp. 189–239). Springer Publishing Company.

[R63] James SA (1994). John Henryism and the health of African-Americans. Culture, Medicine and Psychiatry, 18(2), 163–182. 10.1007/BF01379448 7924399

[R64] Juster R-P, McEwen BS, & Lupien SJ (2010). Allostatic load biomarkers of chronic stress and impact on health and cognition. Psychophysiological Biomarkers of Health, 35(1), 2–16. 10.1016/j.neubiorev.2009.10.002 19822172

[R65] Kirkland JL, Stout MB, & Sierra F (2016). Resilience in aging mice. The Journals of Gerontology: Series A, 71(11), 1407–1414. 10.1093/gerona/glw086 PMC5865545 27535963

[R66] Kornienko O, Santos CE, Seaton EK, Davila M, & Garner PW (2023). Racial discrimination experiences and friendship network dynamics among Black and Latinx youth. Journal of Youth and Adolescence, 52(4), 685–700. 10.1007/s10964-023-01746-1 36807230

[R67] Kwon E, Metzger I, & Kogan SM (2022). Racial discrimination and conduct problems among Black American youth: The moderating effect of ethnic racial socialization. Journal of Adolescent Health, 71(4), 488–493. 10.1016/j.jadohealth.2022.05.007 PMC13020752 35779997

[R68] Logan JG, & Barksdale DJ (2008). Allostasis and allostatic load: Expanding the discourse on stress and cardiovascular disease. Journal of Clinical Nursing, 17(7b), 201–208. 10.1111/j.1365-2702.2008.02347.x 18578796

[R69] Major B, Kaiser CR, & McCoy SK (2003). It’s not my fault: When and why attributions to prejudice protect self-esteem. Personality and Social Psychology Bulletin, 29(6), 772–781. 10.1177/0146167203029006009 15189632

[R70] Major JLL (2020). Racial discrimination, ethnic-racial socialization, depression, and educational attainment in a longitudinal study of African American youth. Iowa State University Digital Repository.

[R71] Masten AS (2024). Emergence and evolution of developmental resilience science over half a century. Development and Psychopathology, 1–9. Cambridge Core. 10.1017/S0954579424000154 38456302

[R72] McEwen BS, & Seeman T (1999). Protective and damaging effects of mediators of stress. Elaborating and testing the concepts of allostasis and allostatic load. Annals of the New York Academy of Sciences, 896(1), 30–47. 10.1111/j.1749-6632.1999.tb08103.x 10681886

[R73] Mezuk B, Abdou CM, Hudson D, Kershaw KN, Rafferty JA, Lee H, & Jackson JS (2013). “White box” epidemiology and the social neuroscience of health behaviors: The environmental Affordances model. Society and Mental Health, 3(2), 79–95. 10.1177/2156869313480892 PMC3820104 24224131

[R74] National Research Council. (2004). Measuring racial discrimination. The National Academies Press. 10.17226/10887

[R75] Neblett EW, White RL, Ford KR, Philip CL, Nguyên HX, & Sellers RM (2008). Patterns of racial socialization and psychological adjustment: Can parental communications about race reduce the impact of racial discrimination? Journal of Research on Adolescence, 18(3), 477–515. 10.1111/j.1532-7795.2008.00568.x

[R76] Nelson SC, Syed M, Tran AGTT, Hu AW, & Lee RM (2018). Pathways to ethnic-racial identity development and psychological adjustment: The differential associations of cultural socialization by parents and peers. Developmental Psychology, 54(11), 2166–2180. 10.1037/dev0000597 30265036

[R77] Nguyen AW, Chatters LM, Taylor RJ, Aranda MP, Lincoln KD, & Thomas CS (2018). Discrimination, serious psychological distress, and Church-based emotional support among African American men across the life span. The Journals of Gerontology: Series B, 73(2), 198–207. 10.1093/geronb/gbx083 PMC5926995 29106656

[R78] Park M, Choi Y, Yasui M, & Hedeker D (2021). Racial discrimination and the moderating effects of racial and ethnic socialization on the mental health of Asian American youth. Child Development, 92(6), 2284–2298. 10.1111/cdev.13638 34374432 PMC8932491

[R79] Pew Research Center. (2019). 3. The role of race and ethnicity in Americans’ personal lives. Pew Research Center’s Social & Demographic Trends Project. Retrieved from https://www.pewresearch.org/social-trends/2019/04/09/the-role-of-race-and-ethnicity-in-americans-personal-lives/

[R80] Phinney JS, & Ong AD (2007). Conceptualization and measurement of ethnic identity: Current status and future directions. Journal of Counseling Psychology, 54(3), 271–281. 10.1037/0022-0167.54.3.271

[R81] Raffington L, & Belsky DW (2022). Integrating DNA methylation measures of biological aging into social determinants of health research. Current Environmental Health Reports, 9(2), 196–210. 10.1007/s40572-022-00338-8 35181865

[R82] Reynolds JE, & Gonzales-Backen MA (2017). Ethnic-racial socialization and the mental health of African Americans: A critical review. Journal of Family Theory & Review, 9(2), 182–200. 10.1111/jftr.12192

[R83] Saleem FT, Lambert S, & Rose T (2022). Ethnic–racial socialization as a moderator of associations between discrimination and psychosocial well-being among African American and Caribbean Black adolescents. Cultural Diversity and Ethnic Minority Psychology, 28(2), 145–157. 10.1037/cdp0000521 35099209

[R84] Scott JC, Pinderhughes EE, & Johnson SK (2019). How does racial context matter? Family preparation-for-bias messages and racial coping reported by Black youth. Child Development, 91(5), 1471–1490. 10.1111/cdev.13332 31659748 PMC7188572

[R85] Seol KO, Yoo HC, Lee RM, Park JE, & Kyeong Y (2016). Racial and ethnic socialization as moderators of racial discrimination and school adjustment of adopted and nonadopted Korean American adolescents. Journal of Counseling Psychology, 63(3), 294–306. 10.1037/cou0000120 26479418 PMC4833521

[R86] Simon C (2021). The role of race and ethnicity in parental ethnic-racial socialization: A scoping review of research. Journal of Child and Family Studies, 30(1), 182–195. 10.1007/s10826-020-01854-7

[R87] Sladek MR, Castro SA, & Doane LD (2021). Ethnic-Racial discrimination experiences predict Latinx adolescents’ physiological stress processes across college transition. Psychoneuroendocrinology, 128, 105212. 10.1016/j.psyneuen.2021.105212 33933893

[R88] Sladek MR, Umaña-Taylor AJ, Hardesty JL, Aguilar G, Bates D, Bayless SD, Gomez E, Hur CK, Ison A, Jones S, Luo H, Satterthwaite-Freiman M, & Vázquez MA (2022). “So, like, it’s all a mix of one”: Intersecting contexts of adolescents’ ethnic-racial socialization. Child Development, 93(5), 1284–1303. 10.1111/cdev.13756 35366330

[R89] Smith-Bynum MA, Anderson RE, Davis BL, Franco MG, & English D (2016). Observed racial socialization and maternal positive emotions in African American mother–adolescent discussions about racial discrimination. Child Development, 87(6), 1926–1939. 10.1111/cdev.12562 27211821 PMC5121096

[R90] Stevenson HC, & Arrington EG (2009). Racial/ethnic socialization mediates perceived racism and the racial identity of African American adolescents. Cultural Diversity and Ethnic Minority Psychology, 15(2), 125–136. 10.1037/a0015500 19364199

[R91] Sullivan CM, & O’Keeffe ZP (2017). Evidence that curtailing proactive policing can reduce major crime. Nature Human Behaviour, 1(10), 730–737. 10.1038/s41562-017-0211-5 31024103

[R92] Toomey RB, Shramko M, Flores M, & Anhalt K (2018). Family socialization for racism and heterosexism: Experiences of Latinx sexual minority adolescents and young adults. Journal of Family Issues, 39(13), 3586–3611. 10.1177/0192513X18783807 PMC12382482 40881672

[R93] Ukraintseva S, Arbeev K, Duan M, Akushevich I, Kulminski A, Stallard E, & Yashin A (2021). Decline in biological resilience as key manifestation of aging: Potential mechanisms and role in health and longevity. Mechanisms of Ageing and Development, 194, 111418. 10.1016/j.mad.2020.111418 33340523 PMC7882032

[R94] Umaña-Taylor AJ (2024). Revisiting the conceptualization and measurement of ethnic-racial identity affect: Recommendations for future directions. Child Development Perspectives. 10.1111/cdep.12517

[R95] Umaña-Taylor AJ, & Hill NE (2020). Ethnic–racial socialization in the family: A decade’s advance on precursors and outcomes. Journal of Marriage and Family, 82(1), 244–271. 10.1111/jomf.12622

[R96] Wang M-T, Henry DA, Smith LV, Huguley JP, & Guo J (2019a). Parental ethnic-racial socialization practices and children of color’s psychosocial and behavioral adjustment: A systematic review and meta-analysis. American Psychologist, 75(1), 1–22. 10.1037/amp0000464 31058521

[R97] Wang M-T, & Huguley JP (2012). Parental racial socialization as a moderator of the effects of racial discrimination on educational success among African American adolescents. Child Development, 83(5), 1716–1731. 10.1111/j.1467-8624.2012.01808.x 22717004

[R98] Wang M-T, Smith LV, Miller-Cotto DA, & Huguley JP (2019b). Parental ethnic-racial socialization and children of color’s academic success: A meta-analytic review. Child Development, 91(3). 10.1111/cdev.13254 31099030

[R99] Wheaton B (1985). Models for the stress-buffering functions of coping resources. Journal of Health and Social Behavior, 26(4), 352–364. 10.2307/2136658 4086758

[R100] Yip T, Wang Y, Mootoo C, & Mirpuri S (2019). Moderating the association between discrimination and adjustment: A meta-analysis of ethnic/racial identity. Developmental Psychology, 55(6), 1274–1298. 10.1037/dev0000708 30907605 PMC6557142

[R101] Zeiders KH, Causadias JM, & White RMB (2017). The health correlates of culture: Examining the association between ethnic-racial identity and diurnal cortisol slopes. Journal of Adolescent Health, 62(3), 349–351. 10.1016/j.jadohealth.2017.09.020 29203357

[R102] Zeiders KH, Umaña-Taylor AJ, Martinez-Fuentes S, Updegraff KA, Douglass Bayless S, & Jahromi LB (2021). Latina/o youths’ discrimination experiences in the U.S. Southwest: Estimates from three studies. Applied Developmental Science, 25(1), 51–61. 10.1080/10888691.2018.1527695 33716491 PMC7953829

